# Latent transition analysis of resilience in breast cancer patients and its association with sense of meaning in life: a longitudinal follow - up study

**DOI:** 10.3389/fonc.2026.1769713

**Published:** 2026-06-11

**Authors:** Huiping Tian, Yue Wang, Song Guo

**Affiliations:** Department of Medical Oncology, Affiliated Hospital of Hebei University, BaoDing, Hebei, China

**Keywords:** breast cancer, latent profile analysis, latent transition analysis, psychological resilience, relevance, sense of meaning in life

## Abstract

**Background:**

Breast cancer is a highly prevalent malignant tumor among women, showing a trend of younger onset. The disease - related stress can easily trigger an existential crisis and reduce the sense of meaning in life. However, the sense of meaning in life and psychological resilience are key psychological resources crucial for disease adaptation. Nevertheless, previous studies have mostly adopted cross - sectional designs, which limits the ability to clarify the dynamic evolution of these constructs.

**Aim:**

To explore the changes of latent categories of resilience over time in breast cancer patients and their correlation with the sense of meaning in life.

**Methods:**

A convenience sampling method was used to recruit 223 breast cancer patients. Measurements using the Connor-Davidson Resilience Scale and the Chinese version of the Meaning in Life Questionnaire were conducted at post-surgery (T1) and 3 months post-surgery (T2).

**Results:**

At T1 and T2, there were two latent profile categories of psychological resilience, namely the low psychological resilience group (C1) and the high psychological resilience group (C2). The results of Latent Transition Analysis showed that 31.48% of breast cancer patients continued to be in the C2 group (persistent low - risk group), 25.25% of breast cancer patients continued to be in the C1 group (persistent high - risk group), 38.63% of breast cancer patients changed from the C1 group to the C2 group (Resilience Enhancing Group), and 5.64% of breast cancer patients changed from the C2 group to the C1 group (resilience decline group). Multivariate Logistic regression analysis showed that compared with the persistent low - risk group, breast cancer patients in the persistent high - risk group (OR = 9.488), the Resilience Enhancing Group (OR = 1.667), and the resilience decline group (OR = 4.899) had an increased risk of low life meaning (all P < 0.001).

**Conclusions:**

The latent change in resilience is significantly related to the meaning in life of breast cancer patients. Early identification and continuous psychological intervention are required to enhance the meaning in life of breast cancer patients.

## Introduction

In 2022, there were approximately 2.3 million new cases and 670,000 deaths from breast cancer worldwide. Breast cancer has become the most prevalent malignant tumor among women globally, accounting for 25% of new cancer cases in women (1, 2). It is projected that by 2050, the number of new global breast cancer cases will increase by 38% to 3.2 million, and deaths will rise by 68% to 1.1 million. Most of this growth will be concentrated in low - and middle - income countries (3, 4). In China, breast cancer also poses a major threat to women’s health. In 2022, there were about 357,200 new breast cancer cases in China, ranking second among female malignant tumors, and approximately 75,000 deaths (5). The incidence of breast cancer in China exhibits obvious regional disparities. The incidence of breast cancer in urban areas is significantly higher than that in rural areas, and the average age of onset is about 10 years earlier than that in European and American countries (6, 7).

Meaning in life refers to an individual’s internal perception and conviction regarding the purpose and value of their own existence. It encompasses the overall understanding of the coherence of the life course, the significance of life events, and the clarity of life goals. It is the core psychological construct that allows people to experience a sense of integrity, value, and direction in life (8). For breast cancer patients, the physical pain caused by the disease, the disruption of social roles, and the profound worry about the uncertainty of the prognosis can easily induce an existential crisis and shake the foundation of their sense of meaning in life (9, 10). Therefore, maintaining or enhancing the sense of meaning in life holds crucial clinical significance. It is not only an important protective factor for enhancing the patient’s psychological resilience when facing the disease and treatment, and buffering negative emotions such as anxiety and depression, but it can also stimulate the patients’ internal motivation to reconstruct their life narrative and discover the unique meaning of suffering in the context of the disease dilemma. Subsequently, this can be translated into positive behaviors, such as actively participating in treatment, improving the quality of life, and even potentially influencing the survival outcome (11, 12). Therefore, the sense of meaning in life is regarded as the core resource in the psychosocial rehabilitation process of breast cancer patients. Its cultivation and support should be systematically integrated into the entire - course management, ranging from diagnosis and treatment to long - term rehabilitation.

Psychological resilience refers to the dynamic ability of individuals to effectively adapt, maintain or restore their psychological function, and even achieve personal growth when facing major adversity, trauma, or chronic stress. Its core structure encompasses three key dimensions (13, 14): resilience, stress resistance, and post - traumatic growth. For breast cancer patients, high resilience not only acts as a crucial protective factor that can effectively buffer the psychological impact of disease diagnosis, treatment side effects, and prognosis uncertainty, but also facilitates positive cognitive reappraisal and the process of meaning - finding. It can assist patients in building or enhancing their sense of meaning in life during adversity, namely, their profound perception of the purpose and value of their own existence. Consequently, it can enhance treatment compliance, improve the quality of life, and may have a positive influence on long - term survival outcomes (15, 16).

However, previous studies in this field have been largely constrained by cross-sectional designs, remaining confined to static correlational analyzes that fail to capture the dynamic evolution and predictive relationships between variables. To overcome these limitations, this study employs longitudinal dynamic modeling to achieve the following specific objectives:

First, utilizing Latent Profile Analysis (LPA), we aim to identify and characterize heterogeneous subgroups of psychological resilience based on postoperative baseline data. This approach moves away from averaging assumptions to precisely delineate distinct latent profiles of psychological adaptability among breast cancer patients. Second, adopting Latent Transition Analysis (LTA), we longitudinally track the developmental trajectories of these resilience subgroups from the postoperative period to three months post-surgery. By quantifying transition probabilities between latent states, we seek to elucidate the patterns of stability and change governing psychological resilience during the early rehabilitation phase. Third, from a dynamic perspective, we investigate the impact of latent state transitions—such as positive shifts from low to high resilience—on concurrent levels of meaning in life. This analysis aims to clarify the longitudinal predictive relationship between the dynamic evolution of resilience and the reconstruction of meaning in life. Ultimately, based on empirical evidence, this study seeks to provide a theoretical foundation for developing targeted psychological interventions tailored to specific resilience trajectories, thereby fostering the restoration of meaning in life among breast cancer survivors.

## Subjects and methods

### Subjects

From January 2024 to December 2024, convenience sampling was used to select 223 breast cancer patients admitted to our hospital as the research objects.

#### Inclusion criteria

Patients met the clinical diagnostic criteria of breast cancer and were confirmed by postoperative pathology (17).

Patients with an expected survival time ≤ 6 months; Those in the terminal stage who require palliative care focused on symptom relief; Or those with pathologically or radiologically (via CT, MRI, or PET - CT) confirmed local recurrence or distant metastasis (e.g., to the lung, liver, bone, or brain), or a clinical diagnosis of recurrent or metastatic breast cancer (18).

With basic understanding and communication skills, all patients signed informed consent.

#### Exclusion criteria

Patients with an expected survival time ≤ 6 months;

Those in the terminal stage who require palliative care focused on symptom relief;

Or those with pathologically or radiologically (via CT, MRI, or PET - CT) confirmed local recurrence or distant metastasis (e.g., to the lung, liver, bone, or brain), or a clinical diagnosis of recurrent or metastatic breast cancer (18).

Patients who failed to complete 2 longitudinal measurements due to death or other reasons during follow - up.

Before the initiation of the study, a written application was submitted to the bioethics committee of this hospital for authorization to conduct the clinical investigation (number: 2023 - 12 - 0025). This study was conducted in full accordance with the principles of the Declaration of Helsinki (19).

(1) $$\text{n}=\frac{2}{{\text{δ}}^{2}}[{\text{σ}}_{\text{μ}}^{2}+\frac{1+(\text{K}-1){\text{ρ}}_{\text{c}}}{\text{K}}{\text{σ}}_{\text{e}}^{2}]{\left({\text{U}}_{\text{α}/2}+{\text{U}}_{\text{β}}\right)}^{2}$$

According to the repeated-measures sample-size formula (Equation 1) and the pilot study , which required a sample size of at least 164 in combination with a 10% dropout rate from longitudinal surveys, the minimum sample size is calculated as 164 ÷ (1 − 10%) = 183. All subjects volunteered to participate in this study and signed informed consent.

### Survey instrument

#### General information questionnaire

The general information questionnaire was self - compiled by the researchers. It includes the patient’s age, education level, residence, living style, economic income, marital status, tumor stage, whether breast - conserving surgery was performed, postoperative chemotherapy, and so on.

#### Resilience scale

The scale was developed by Wagnild et al (20). and translated into Chinese by Ni Qianyu et al (21). It consists of a total of 14 items, including two dimensions: personal ability and acceptance of self and life. The items are scored using a Likert 7 - point scale, ranging from “completely disagree” to “completely agree”, with scores from 1 to 7 points. The total score range is from 14 to 98 points. The higher the score, the better the psychological resilience. The Cronbach’s α coefficients of the two measurements of the scale in this study were 0.857 and 0.840.

#### The Chinese version of meaning in life questionnaire

The sense of meaning in life refers to an individual’s subjective perception and experience regarding the purpose, value, and significance of their existence. It encompasses both the perception of meaning that has already been found (Presence of Meaning) and the active process of searching for meaning (Search for Meaning), reflecting an individual’s psychological cognition and pursuit of life values. The scale was translated into Chinese by Chinese scholar Liu Sisi et al (22). The C-MLQ consists of two sub-dimensions: Presence of Meaning (5 items) and Search for Meaning (4 items). The Presence of Meaning subscale measures the degree to which an individual currently perceives their life to have meaning, purpose, and value. In contrast, the Search for Meaning subscale assesses the extent to which an individual is actively striving to find meaning and purpose in their life. Each item is rated from “completely disagree” to “completely agree”, and the total score ranges from 9 to 63. The higher the score, the stronger the sense of meaning in life. A score of 9–27 points indicates a low level of meaning in life. A score of 28–45 indicates a moderate level of meaning in life. A score of 46 to 63 indicates a high level of meaning in life. The Cronbach’s α coefficients of the two measurements of the scale in this study were 0.910 and 0.828.

### Data collection methods

Before conducting the survey, all researchers participating in it were trained. After obtaining the consent of hospitals and patients, a questionnaire survey was carried out, and the general - information questionnaire was retrieved from the patients’ medical records. Previous studies have indicated that the psychological changes of breast - cancer patients tend to stabilize after 3 months. Therefore, data from the Connor - Davidson Resilience Scale and the Meaning in Life Assessment Scale were collected at two time points: postoperative/stable disease (T1) and 3 months after surgery (T2) (23). The data at the T1 time point were collected through face - to - face surveys in the ward of breast surgery, while the data at the T2 time point were obtained by researchers through outpatient follow - up or telephone/WeChat follow - up. To ensure patient privacy, the survey was conducted in a confidential environment, and the questionnaire was administered anonymously. To ensure transparency regarding factors potentially influencing adherence, we have explicitly stated that all participants received a modest token of appreciation (a hygiene kit valued at approximately 30 RMB) upon completion of each survey wave.

### Statistical methods

SPSS 26.0 software was employed for descriptive statistics and correlation analysis. Enumeration data were presented as the number of cases and percentages. Measurement data following a normal distribution were expressed as the mean ± standard deviation. The chi-square test was utilized to compare different general demographic characteristics, and the Harman single-factor test was applied for the common method deviation test. Mplus 8.3 software was used for latent profile analysis and latent transition analysis, and the model fit test indicators were compared. The model fit tests included the Akaike information criterion (AIC), Bayesian information criterion ((BIC), sample-corrected BIC (aBIC), Entropy, Lo-Mendell-Rubin adjusted likelihood ratio test (LMR), and bootstrap-based likelihood ratio test (BLRT). The smaller the AIC, BIC, and aBIC results, the better the model fit. The value of Entropy ranges between 0 and 1, and generally, Entropy > 0.8 is required for high explanatory power. Univariate Logistic regression was used to analyze the relationship between the transition mode of resilience and the sense of meaning in life, and multivariate stepwise regression was used to control the confounding effect. A p - value < 0.05 was considered statistically significant.

## Results

### General information of the respondents

**Figure 1:**
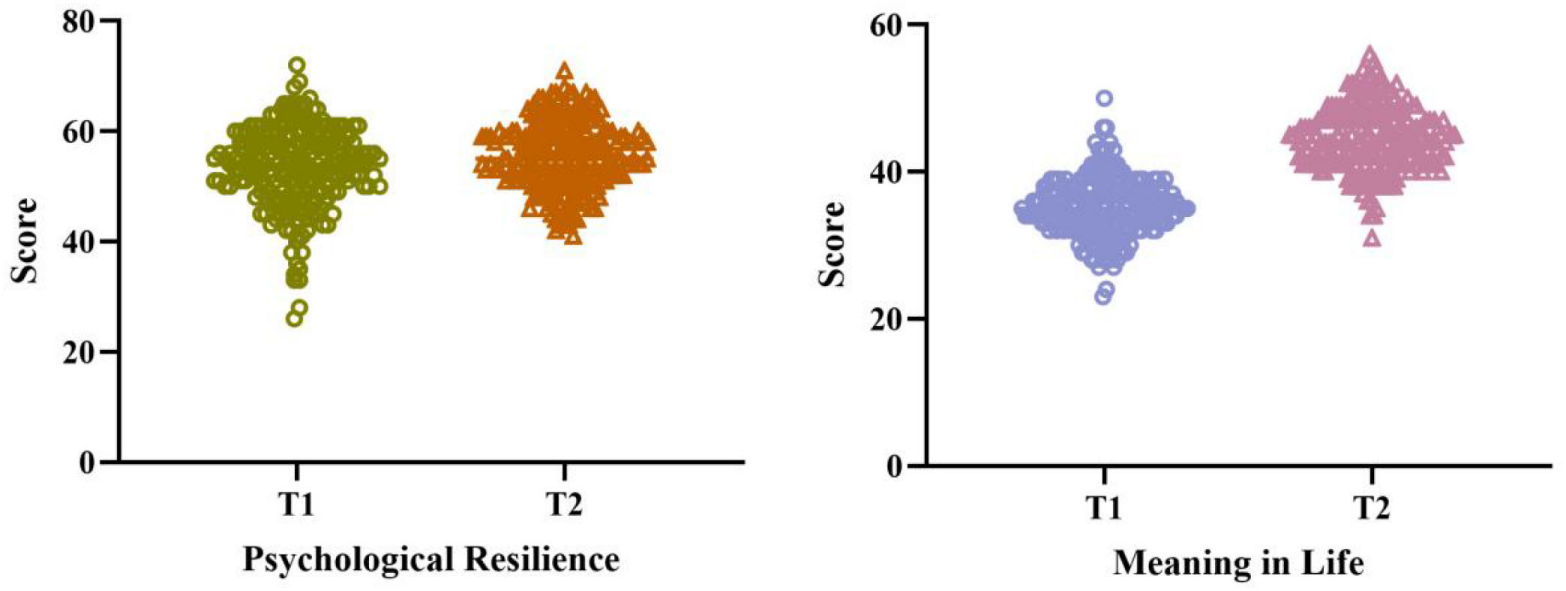
Distributions of scores for psychological resilience and meaning in life.

Three cases were lost to follow - up, and 220 valid questionnaires were collected, with an effective rate of 98.65%. The basic information at T1 is shown in **Table 1**. Distributions of scores for the Resilience Scale (RS-14) and the Chinese Version of the Meaning in Life Questionnaire (C-MLQ) are presented in **Figure 1**.

**Table 1:** General information of the subjects (*n* = 220).

Items	Categories	n	%	Items	Categories	n	%
Age (years)	< 45	53	24.09	Style of residence	Living alone	16	7.27
	45 ~ 59	84	38.18		Family residence	204	92.73
	≥60	83	37.73	Occupation	Enterprises and institutions	49	22.27
Place of residence	Town	107	48.64		Individual	41	18.64
	Rural	113	51.36		Clerk/staff	111	50.45
Education level	Junior high school and below	92	41.82		no	19	8.64
	High school	84	38.18	Tumor staging	I	63	28.64
	Specialist and above	44	20.00		II	76	34.55
Monthly household income	Low income	66	30.00		III~	81	36.82
	Middle income	71	32.27	Breast conserving or not	Yes	128	58.18
	​Upper-middle income	51	23.18		No	92	41.82
	High income	32	14.55	Postoperative chemotherapy	Yes	90	40.91
Marital status	unmarried	8	3.64		No	130	59.09
	Married	199	90.45				
	Divorced	13	5.91				

### Common method bias test

To test for common method bias in the longitudinal survey data, the samples collected at both time points (T1, T2) were subjected to the Harman single - factor test. The results showed that the number of factors with eigenvalues greater than 1 in the T1 and T2 data was 11 and 10, respectively. Moreover, the proportion of variance explained by the first common factor was 16.24% and 21.28%, respectively, which were lower than the critical standard of 40% (24). This indicates that there was no serious common method bias in this study.

### Comparison of meaning in life scores of breast cancer patients at T1 and T2

The score of meaning in life of breast cancer patients at T1 was correlated with age (χ² = 9.815, P = 0.044), family monthly income (χ² = 12.949, P = 0.044), living style (χ² = 9.979, P = 0.007), tumor stage (χ² = 10.864, P = 0.028), and whether they received breast - conserving therapy (χ² = 8.734, P = 0.007). The score of meaning in life of breast cancer patients at T1 was also significantly different from that at T2 (P = 0.013). The scores of meaning in life at T2 were significantly different in terms of family monthly income (χ² = 17.367, P = 0.008), living style (χ² = 9.329, P = 0.009), and postoperative chemotherapy (χ² = 11.419, P = 0.003), as shown in **Table 2**.

**Table 2:** Comparison of meaning in life scores of breast cancer patients at T1 and T2 (*n* = 220).

Variables	T1 Sense of meaning in life score			χ^2^	P-value	T2 Sense of meaning in life score			χ^2^	P-value
	9-27	28 ~ 45	46 ~ 63			9-27	28 ~ 45	46 ~ 63		
Age (years)				9.815	0.044				7.789	0.100
< 45	24	23	6			13	28	12		
45 ~ 59	23	41	20			15	55	14		
≥60	19	48	16			17	39	27		
Marital status				4.252	0.373				4.805	0.308
unmarried	4	2	2			3	3	2		
Married	56	105	38			37	114	48		
Divorced	6	5	2			5	5	3		
Education				1.526	0.822				5.617	0.230
Junior high school and below	24	51	17			15	52	25		
High school	28	40	16			21	49	14		
College or above	14	21	9			9	21	14		
Place of residence				0.782	0.676				0.009	0.996
Town	35	53	19			22	59	26		
Rural	31	59	23			23	63	27		
Monthly household income				12.949	0.044				17.367	0.008
Low income	29	25	12			21	33	12		
Middle income	21	35	15			16	40	15		
​Upper-middle income	11	30	10			5	34	12		
High income	5	22	5			3	15	14		
Occupation				1.104	0.981				4.333	0.632
Enterprises and institutions	16	24	9			10	28	11		
Individual	11	22	8			12	20	9		
Clerk/staff	34	57	20			18	63	30		
no	5	9	5			5	11	3		
Style of residence				9.979	0.007				9.329	0.009
Living alone	10	6	0			8	6	2		
Family living	56	106	42			37	116	51		
Tumor staging				10.864	0.028				4.774	0.311
I	14	29	20			11	31	11		
II	22	42	12			15	40	21		
III~	30	41	10			19	51	11		
Breast conserving or not				8.734	0.013				2.009	0.366
Yes	30	67	31			22	74	32		
No	36	45	11			23	48	21		
Postoperative chemotherapy				3.547	0.170				11.419	0.003
Yes	33	43	14			27	49	14		
No	33	69	28			18	73	39		

### Latent profile analysis of resilience of breast cancer patients

**Figure 2:**
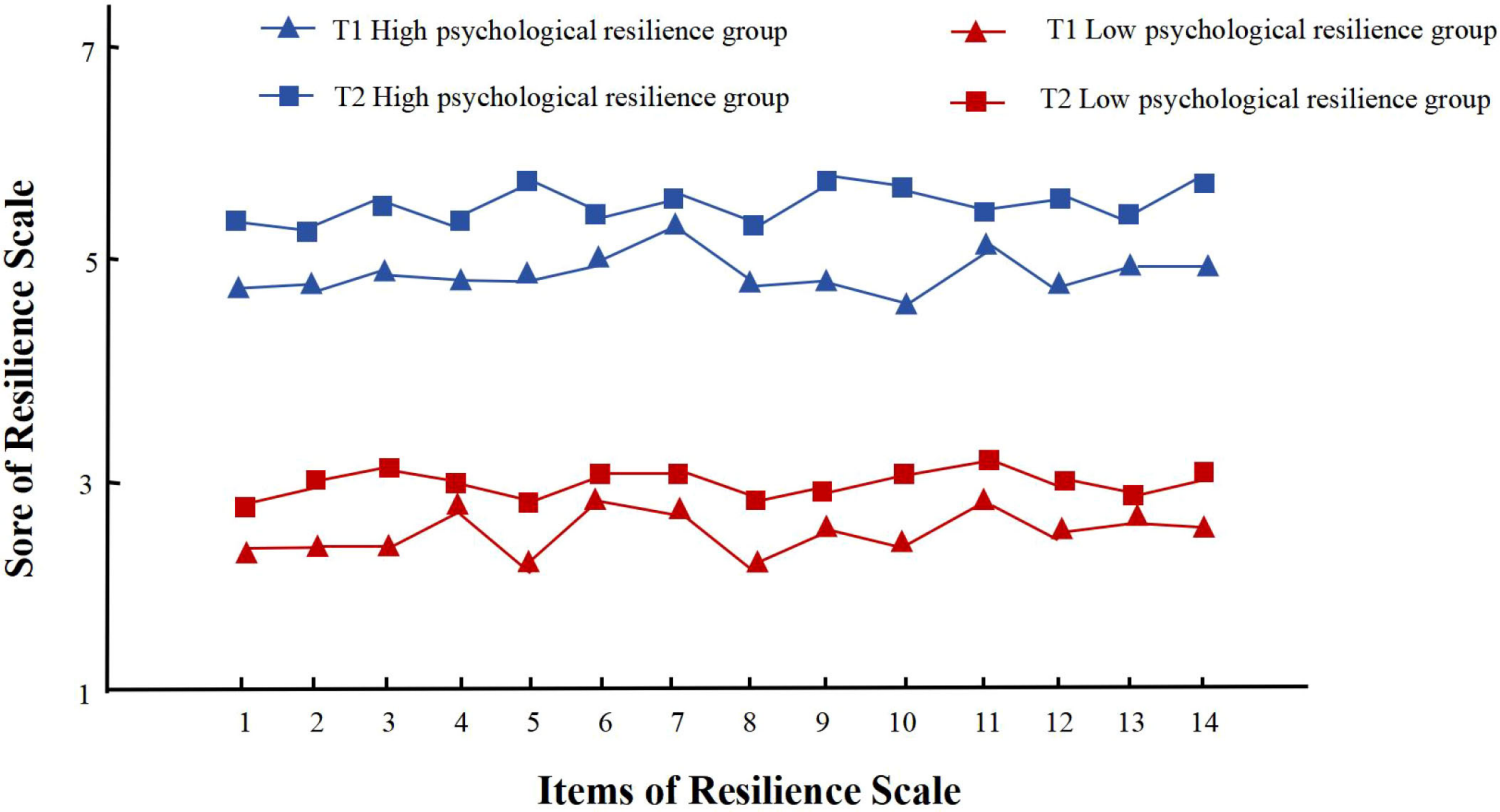
Line plot of latent profile analysis of psychological resilience in breast cancer patients.

In this study, based on the psychological resilience scores, the scores of 14 items were chosen as evaluation indicators. Beginning with the baseline model (where the number of categories was set to 1), models containing 1 to 4 latent profiles were successively fitted and analyzed. The specific data are presented in **Table 3**. At T1 and T2, if the LRT and BLRT were statistically significant and Entropy > 0.8, it indicated that the classification results were highly reliable. Two-category models were ultimately selected in this study. Class 1 was characterized by low overall resilience scores (T1: 2.1–2.8 points per item; T2: 2.8–3.1 points per item), whereas Class 2 exhibited high overall resilience scores (T1: 4.4–5.1 points per item; T2: 5.1–5.7 points per item). Consequently, Class 1 and Class 2 were designated as the Low Resilience Group and the High Resilience Group, respectively (**Figure 2**).

**Table 3:** Comparison of the fitting parameter indexes of different latent profile models.

Time	Model	AIC	BIC	aBIC	Entropy	LRT	BLRT	Class probability
T1	1	1631.941	1656.801	1633.530				
	2	1589.774	1588.905	1588.123	0.825	0.005	< 0.001	0.65/0.35
	3	1586.122	1575.819	1567.206	0.867	0.079	< 0.001	0.24/0.48/0.28
	4	1444.063	1478.231	1419.894	0.882	0.118	0.197	0.25/0.10/0.23/0.42
T2	1	1644.276	1697.247	1638.857				
	2	1518.922	1516.284	1524.933	0.902	0.000	< 0.001	0.44/0.56
	3	1417.988	1457.740	1429.850	0.914	0.065	< 0.001	0.27/0.48/0.25
	4	1312.556	1380.844	1325.887	0.931	0.144	< 0.001	0.25/0.19/0.23/0.33

### Latent transition analysis of psychological resilience of breast cancer patients

Latent transition analysis was used to explore the transformation of the latent profile of psychological resilience among breast cancer patients at time points T1 and T2. The results showed that 55.73% of breast cancer patients maintained the probability of their original latent state. Specifically, 31.48% of breast cancer patients continuously remained in the high psychological resilience group, so they were named the persistent low - risk group; 25.25% of breast cancer patients continuously remained in the low psychological resilience group, so they were named the persistent high - risk group; 38.63% of breast cancer patients changed from the low psychological resilience group to the high psychological resilience group, and they were named the Resilience Enhancing Group. A total of 5.64% of breast cancer patients changed from the high psychological resilience group to the low psychological resilience group, and they were named the Decreased Resilience Group, as shown in **Table 4**.

**Table 4:** T1-T2 transition probability of psychological resilience in breast cancer patients (*n* = 220).

Items		T2	
		C1: high psychological resilience group	C2: low psychological resilience group
T1	C1: High psychological resilience group	31.48	5.64
	C2: Low psychological resilience group	38.63	24.25

### Influence of resilience transition mode of breast cancer patients on sense of meaning in life

Accumulating evidence indicates that psychological resilience serves as a key predictor of meaning in life. Specifically, in breast cancer patients, enhanced resilience facilitates the reconstruction of life goals, thereby strengthening the presence of meaning. Consequently, grounded in this theoretical rationale and empirical support from existing literature, we incorporated the resilience transition patterns as the core independent variable in our regression model (25, 26). The influence of the psychological resilience transition mode of breast cancer patients on their sense of meaning in life was further analyzed. Univariate and multivariate regression analyzes were conducted with the persistent low - risk group as the reference group. Taking the psychological resilience transition mode of breast cancer patients as the independent variable and the sense of meaning in life (9 - 27 = 1, 28 - 45 = 2, 46 - 63 = 3) as the dependent variable, in univariate logistic regression analysis, compared with the continuous low - risk group, breast cancer patients in the persistent high - risk group, the Resilience Enhancing Group, and the Resilience Decreasing Group had an increased risk of low meaning in life (all P < 0.001).

In Model 2 (controlling for general demographic characteristics) and Model 3 (controlling for general demographic characteristics and T1 meaning in life), breast cancer patients in the Resilience Enhancing Group and the Resilience Decreasing Group had an increased risk of low meaning in life (all P < 0.05), but the risk was the lowest in the Resilience Enhancing Group (OR = 1.654), as shown in **Table 5**.

**Table 5:** Association between resilience and meaning in life in breast cancer patients.

Patterns of transition	*B*	*SE*	OR	95%CI	P-value
Model 1					
Persistent low-risk group	–	–	–	–	
Persistent high-risk group	2.914	0.449	18.430	7.644 ~ 44.436	< 0.001
Resilience Enhancing Group	0.933	0.220	2.542	1.652 ~ 3.913	< 0.001
Decreased resilience group	1.623	0.248	5.068	3.177 ~ 8.241	< 0.001
Model Two					
Persistent low-risk group	–	–	–	–	
Persistent high-risk group	2.689	0.313	14.717	7.969 ~ 27.180	< 0.001
Resilience Enhancing Group	0.897	0.240	2.452	1.532 ~ 3.925	< 0.001
Decreased resilience group	1.344	0.351	3.834	1.927 ~ 7.629	< 0.001
Model III					
Persistent low-risk group	–	–	–	–	
Persistent high-risk group	2.250	0.355	9.488	4.731 ~ 19.026	< 0.001
Resilience Enhancing Group	0.511	0.194	1.667	1.140 ~ 2.438	0.011
Decreased resilience group	1.589	0.356	4.899	2.438 ~ 9.843	< 0.001

Model 1: Logistic regression; Model 2: confounding factors of age, marriage, education level, living style, residence, occupation, family income, tumor stage, breast-conserving therapy and chemotherapy were controlled. Model 3: age, marriage, education level, living style, residence, occupation, family income, tumor stage, breast conservation, chemotherapy and T1 time meaning in life were controlled.

## Discussion

The differences in postoperative meaning in life among breast cancer patients reflect that the disease and its treatment, as a major life - stressful event, interact with the patients’ social demographic characteristics, disease severity, and treatment mode to shape their psychological adaptation trajectory. The significant age difference in the sense of meaning in life immediately after surgery may be due to the different developmental tasks and social roles faced by patients of different ages. Younger patients may be in the critical period of career climbing, family building, or raising young children. The impact of cancer diagnosis on their identity and future planning is more severe, which is likely to lead to a stronger sense of meaninglessness (27). With the accumulation of life experience, middle - aged and elderly patients may have a more thorough understanding of life, which helps them maintain a relatively stable sense of meaning (28). As a persistent and significant influencing factorWhether in the postoperative period or 3 months after surgery, a patient’s financial status is directly related to their ability to obtain adequate medical resources, reduce anxiety about treatment costs, and impacts their quality of life during rehabilitation. Financial stress itself consumes psychological resources, making it more difficult for patients to focus on finding positive meaning in life (29, 30). The density of social support reflected by living style is crucial. People who live with their families are more likely to obtain timely emotional comfort and daily care. This close social bond serves as a buffer to cope with the challenges of the disease. In contrast, people who live alone are more likely to fall into loneliness and helplessness, which affects the construction of their sense of meaning (31).

The higher the tumor stage, the more serious the invasion of the disease into the body, the stronger the uncertainty about the future. Moreover, patients need to endure greater physiological pain and fear of prognosis. These directly challenge their original beliefs about the control and value of life (32, 33). Whether to accept breast - conserving surgery has a significant impact on patients’ sense of meaning, which goes beyond the simple choice of surgical methods and deeply touches women’s body image, self - identity, and gender identity. Breast - conserving surgery helps maintain the sense of body integrity and self - esteem to a certain extent and reduces negative feelings such as “incompleteness” and “devaluation” caused by treatment. Therefore, it preserves an important psychological space for finding the positive meaning of life (34, 35). By 3 months after surgery, the effect of the treatment mode (such as postoperative chemotherapy) is highlighted. The side effects of chemotherapy, such as alopecia, vomiting, and fatigue, not only continue to impair physical function, but also the visible external changes, such as alopecia, constantly remind the patient of her role as a patient, which may cause existential conf A confusion of “why me”. If patients avoid rather than face challenges, they will further aggravate their sense of meaninglessness (36, 37).

This study identified four transition patterns of resilience in breast cancer patients based on latent transition analysis, and their distribution showed significant heterogeneity. 38.63% of the patients changed from the low psychological resilience group to the high psychological resilience group (Resilience Enhancing Group), and their positive transformation may benefit from an effective social support system, a positive coping style, and interventions that may be targeted to enhance psychological resilience resources. On the contrary, 5.64% of the patients changed from the high psychological resilience group to the low psychological resilience group (Decreased Resilience Group). The loss of resilience may be related to continuous significant stress, such as recurrent treatment side - effects, an increased economic burden, a lack of sufficient social connection, or exhaustion of support resources. Resources, and failure to effectively prevent “catastrophizing” thinking (38, 39)This suggests that clinical medical staff should implement dynamic assessment and stratified intervention strategies. For the “persistent high - risk group”, multi - dimensional support should be strengthened, especially to alleviate economic toxicity and manage symptoms. For the Resilience Enhancing Group, the positive transformation experience should be consolidated to form a “resilience flywheel”. For the resilience decline group, it is necessary to identify and intervene in their risk factors early to prevent further loss of resilience. At the same time, promoting positive social comparison and sharing of successful experiences among patients may provide a reference for groups with different trajectories to improve resilience.

Logistic regression analysis revealed that compared with the persistent low - risk group, breast cancer patients in the persistent high - risk group, the Resilience Enhancing Group, and the decrease. The low resilience group had a significantly increased risk of low life meaning, which was due to the cumulative effect and dynamic interaction of multi - dimensional risk factors. Patients in the persistent high - risk group usually face stable risk factors such as low social support, low economic income, and negative coping styles. These factors together limit their ability to obtain psychological resources and hinder the occurrence of post - traumatic growth, resulting in a persistently low sense of meaning in life (40). Although patients in the decreased resilience group had high psychological resilience at the beginning, they may encounter new stressors during treatment, such as aggravation of chemotherapy side effects, appearance of economic toxicity, or weakening of social support. Their psychological resources are continuously consumed, and their intrinsic positive coping ability is not enhanced simultaneously, resulting in a significant reduction in the sense of meaning in life with the decline. This text seems to be incomplete as it ends abruptly. However, I’ll proofread and refine the provided part:

The concept of resilience. Although the resilience level of patients in the Resilience Enhancing Group showed positive changes, their sense of meaning in life was still at risk. This may be because this group is in the dynamic process of psychological adaptation, and the construction speed of their sense of meaning lags behind the improvement of resilience. Or it could be because they still need to cope with the body - image challenges and symptom burden brought by the disease in the early stage of transformation (41).

This risk model suggests that clinical intervention needs to go beyond static risk assessment and implement dynamic, stratified, and precise support strategies. For the persistent high - risk group, we should focus on building a stable social support network and promoting positive coping strategies. For the group with decreased resilience, it is necessary to identify early the risk signals such as economic burden and symptom deterioration and carry out active intervention to prevent… Exhaustion of psychological resources. For the Resilience Enhancing Group, on the basis of the improvement of resilience, the guidance and cultivation of the meaning of life should be strengthened simultaneously. For example, meaning perception can be promoted through mindfulness intervention to ensure that the improvement of psychological resilience can effectively translate into substantial growth in the meaning of life.

## Limitations

This study has several limitations. First, the use of convenience sampling from a single center limits the representativeness of the sample, potentially introducing selection bias and affecting the generalizability of the findings. Second, the assessment was restricted to only two time points (postoperative baseline and 3 months postoperatively), which may not fully capture the long-term dynamic trajectories of psychological resilience and meaning in life throughout the entire treatment and rehabilitation phases. Third, reliance on self-report measures may be susceptible to common method bias and social desirability effects, and it lacks consideration of objective clinical indicators such as complications and specific treatment details. Finally, despite the longitudinal design, the study cannot fully establish a causal relationship between psychological resilience and meaning in life. Additionally, the absence of assessments for hopelessness and expectation represents a notable limitation, yet it also provides a valuable direction for future research. Future studies should employ multicenter designs with extended follow-up periods and integrate mixed-methods approaches to validate these findings.

## Conclusion

31.48% of breast cancer patients remained in the high psychological resilience group, 25.25% of breast cancer patients remained in the low psychological resilience group, 38.63% of breast cancer patients shifted from the low psychological resilience group to the high psychological resilience group, and 5.64% of breast cancer patients shifted from the high psychological resilience group to the low psychological resilience group. Compared with the persistent low - risk group, breast cancer patients in the persistent high - risk group, the Resilience Enhancing Group, and the decreased resilience group had an increased risk of low life meaning.

## Data Availability

The original contributions presented in the study are included in the article/supplementary material. Further inquiries can be directed to the corresponding author.
